# African colorectal cancer burden in 2022 and projections to 2050

**DOI:** 10.3332/ecancer.2024.1780

**Published:** 2024-09-26

**Authors:** Mounoume Lobe Irma Louise Virginie, Qiu Zhao, Lan Liu

**Affiliations:** 1 Department of Gastroenterology, Zhongnan Hospital of Wuhan University, Wuhan 430071, China; 2 Hubei Clinical Center & Key Laboratory of Intestinal and Colorectal Disease, Wuhan 430071, China

**Keywords:** Africa, colorectal cancer, burden, projections

## Abstract

**Introduction:**

The burden of colorectal cancer (CRC) is on a rapid increase on the African continent, yet grossly under reported. Herein, we provide and updated estimates of CRC burden (incidence and mortality) across Africa as of 2022, and make crucial predictions to 2050.

**Methods:**

We gathered information on CRC incidence and mortality from the GLOBOCAN 2022 database, which covers 185 countries. The age-standardised incidence and mortality rates (ASRs) per 100,000 person-years were determined. Cases and deaths up to 2050 were estimated using 2022 incidence and mortality rates.

**Results:**

In 2022, an estimated 70,428 cases and 46,087 mortalities due to CRC were recorded across the African continent. Africa’s ASRs for CRC incidence and mortality were 8.2 and 5.6 per 100,000 population, respectively, and were highest in North Africa followed by East Africa. At national levels, CRC ranked in the top four of the most commonly diagnosed cancers in more than half (56%) of African countries. ASRs of both incidence and mortality were higher among males than females. New cases are predicted to increase by 139.7% (from 70,428 in 2022 to 168,683 in 2050) at the current incidence rate. Similarly, mortalities will increase by 155.2% (from 46,061 in 2022 to 117,568 in 2050).

**Conclusion:**

CRC remains a major cause of morbidity and mortality in many African countries, and the number of new cases and deaths is predicted to rise significantly by 2050. Efforts to reduce the incidence of preventable CRC cases should be prioritised.

## Introduction

Colorectal cancer (CRC) stands as one of the most prevalent cancers worldwide. As delineated by the 2020 Global Cancer Observatory (GLOBOCAN) report, CRC occurred in more than 1.9 million new patients, and caused 900,000 deaths, ranking third and second globally with reference to incidence and mortality, respectively [1]. The global incidence of this pervasive and debilitating cancer has almost tripled from 1990 to 2020 in 157 countries, while mortality has doubled in 129 countries, imposing a substantial economic burden on the often impoverished populations of the affected countries [2].

Current CRC estimates suggest a dramatic increase to 2.2 million new cases and 1.1 million deaths by 2030 [3] owing to the rapid world’s population growth, aging and rapid urbanization in low and middle income countries (LMICs). Trend analyses of global cancer datasets have unmasked CRC as a marker of demographic, epidemiological and socioeconomic shift, manifesting distinct trends; i.e. exponential increase in various LMICs, and stabilisation and decline in many high-income countries [1, 4]. This underscores the tight linkage between CRC and urban lifestyle.

CRC incidence manifests a predilection for the male gender. 1 in 23 males versus 1 in 25 females have a lifetime risk of developing CRC [5]. While approximately 70% of CRC cases are random, 12%–35% are linked to known genetic and lifestyle factors [6, 7]. While not definitive, contemporary cancer studies posit obesity, smoking, excessive alcohol use, junk diet and physical inactivity as key enablers of CRC development in about 55% of cases [5].

In 2012, members of the World Health Assembly committed to cutting by 25% the non-communicable disease (NCD)-related deaths by 2025 [8]. Similarly, the 2015 United Nations (UN) Sustainable Development Goals advocates for a one-third decrease in premature mortality linked to NCDs by 2030 [9]. To evaluate the efficacy of these ambitious targets, understanding the trends and variations in CRC incidence and mortality is crucial. In this regard, GLOBOCAN data offer a reliable source of information covering the national, regional, continental and global burden of cancers. In this study, we sought to assess the burden of CRC across the African continent as of 2022 and make predictions for 2050. This information is vital for planners, policy makers, government health departments and cancer researchers to facilitate a coordinated evidence-based approach to fighting CRC in Africa.

## Data sources and methods

We obtained the number of new cases of and deaths from CRC (C18–C21) from GLOBOCAN 2022, which encompasses global cancer datasets for 185 countries and territories, categorised by sex, cancer type and age brackets (0–4, 5–9,…, 80–84, 85 and over). Data sources and methods used to compile these cancer estimates for 2022 are described online at the Global Cancer Observatory (GCO) [10]. Corresponding population data for 2022 were obtained from the UN website [11]. GLOBOCAN estimates are obtained and assembled at national levels from the best sources of cancer data available within a given country, and so the validity of all cancer estimates here is directly related to the quality of the country-level source data. Country-specific GLOBOCAN data are mainly obtained from the Incidence in Five Continents (CI5) series that provides the best available data on the incidence of cancer recorded by cancer registries (regional or national) around the world during a specific period of time (typically 5 years) hence giving further credence to the integrity of the data used [10].

We extracted the number of new CRC cases and deaths for the African continent where cancers of the colon (C18), rectum (C19-C20) and anus (C21) were combined to make up CRC (C18-C21). In addition to the number of new cases and deaths, two measures of direct standardisation that allow comparisons between populations adjusted for differences in their age structures are used: age-standardised (incidence and mortality) rates (ASRs) per 100,000 person-years based on the 1966 Segi–Doll World standard population [12] and the cumulative risk of (developing or dying from) cancer before age 75 years, assuming the absence of competing causes of death, expressed as a percentage. Cases, deaths and ASRs of CRC are presented by country, by the five regions of Africa based on UN definitions, and by the UN’s four-tier Human Development Index (HDI) in 2022 based on the UN Development Program’s Human Development Report 2021–22 [13], using the predefined four-tier (low, medium, high and very high HDI). The latter describes a means to assess the burden, the strength of health systems and the ability to report CRC cases and deaths at varying levels of development (low, medium, high and very high HDI).

We predicted the future number of CRC cases and deaths up to the year 2050 based on the medium-variant UN population projections and the current global-level incidence and mortality rates of CRC for 2022. The predicted number of new cancer cases or deaths was computed by multiplying the age-specific incidence or mortality rates for the world for 2022 by the corresponding projected world population estimate. These expected populations differ from that of 2022 in terms of age structure and size [14].

## Results

### Current burden of CRC incidence and mortality in Africa

**Figure 1.**
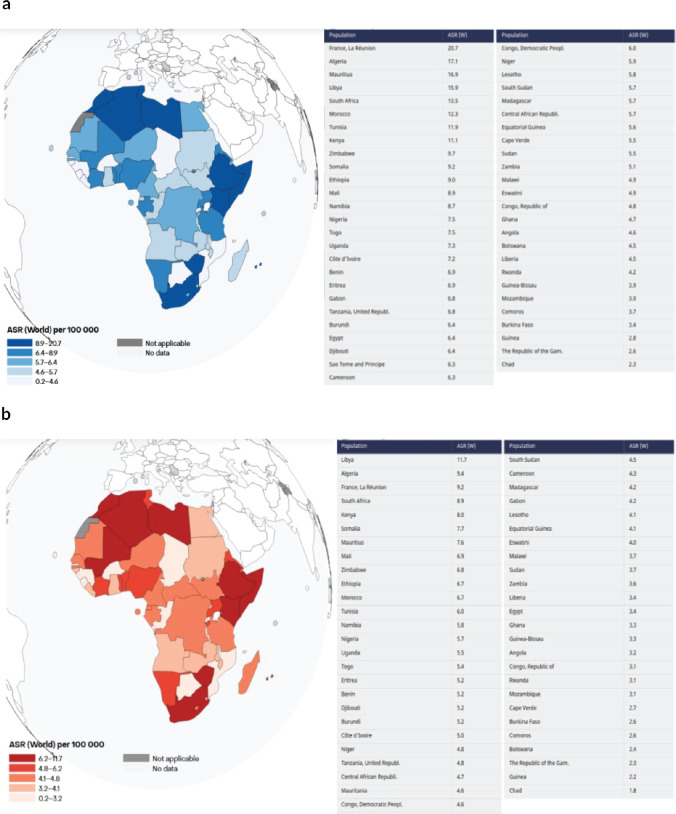
(a): ACR (World) per 100,000, incidence, both sexes, in 2022, colorectum. (b): ACR (World) per 100,000, mortality, both sexes, in 2022, colorectum.

As of 2022, the incidence and mortality of CRC in Africa were estimated at 8.2 and 5.6 per 100,000 population, respectively, with a clear predilection for the male gender (9.1 versus 7.5) Figure 1. Absolute figures revealed an estimated 36,427 colon cancer, 27,645 rectal cancer and 6,356 anal cancer cases recorded across the African continent, summing up to 70,428 CRC cases in 2022. Northern Africa accounted for an outright majority of the cases (33.4%), despite inhabiting only 18.4% of Africa’s population at the time. Regarding morality, most CRC deaths occurred in Eastern Africa (30.0%), trailed closely by Northern Africa (28.4%). Collectively, East and North Africa held more than half of the continent’s CRC incidences and mortalities at 60.9% and 58.4%, respectively, Table 1.

**Figure 2.**
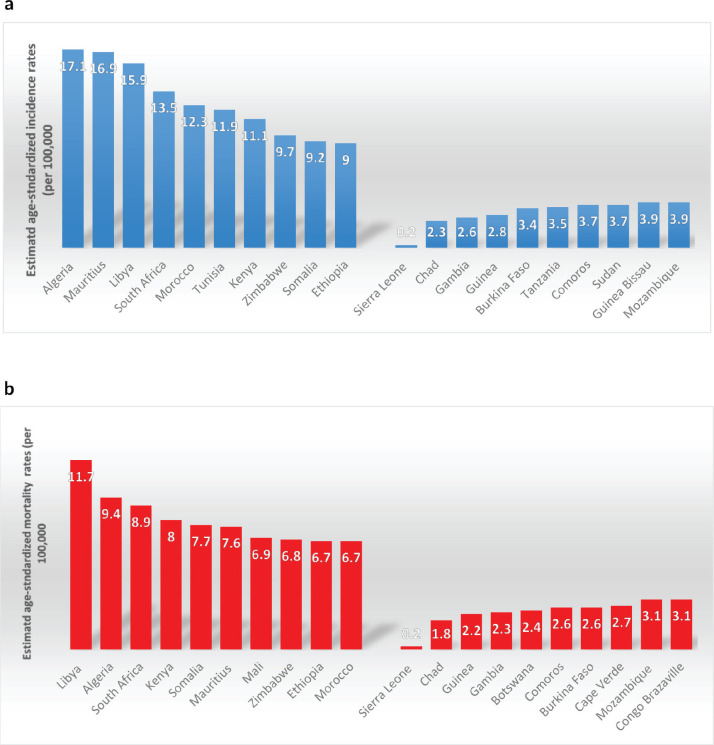
(a): Top ten and bottom ten countries for estimated age-standardised CRC incidence across Africa in 2022. (b): Top ten and bottom ten countries for

**Figure 3.**
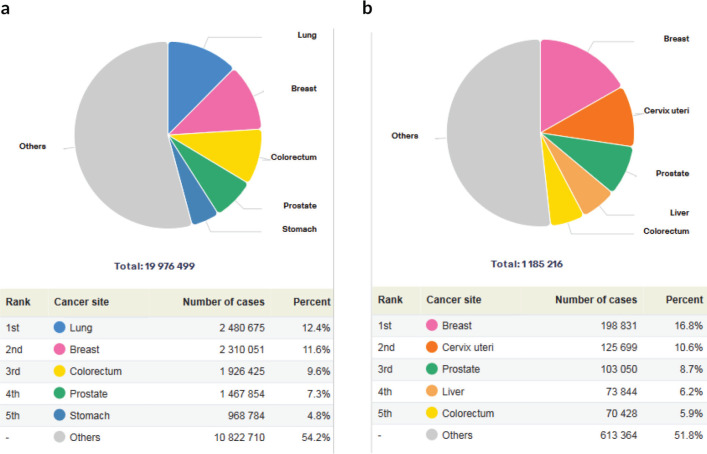
(a): Global incidence and ranking of CRC in 2022. (b): Africa incidence and ranking of CRC in 2022.

National-level analyses divulged wider variations in both incidences and mortalities of CRC. ASR per 100,000 persons was highest in Algeria (17.1%), Mauritius (16.9%) and Libya (15.9%), while ASR for mortality were highest in Libya (11.7%), Algeria (9.4%) and South Africa (8.9%) Figure 2. Figure 3 exhibits the top and bottom ten countries with the highest and lowest estimated ASR incidence and mortality rates across the continent in 2022, while Table 2 compares CRC standardised incidence and mortality rates by continent in 2022. Disparities by gender were apparent, with CRC incidence and mortality higher among males than females in all the regions except Eastern Africa. The incidence M:F ratio ranged from 0.94 in Eastern Africa to 1.25 in Western Africa, while the mortality M:F ratio ranged from 0.92 in Eastern Africa to 1.24 in Western Africa Table 1.

### Ranking of CRC diagnoses and deaths in Africa

**Figure 4.**
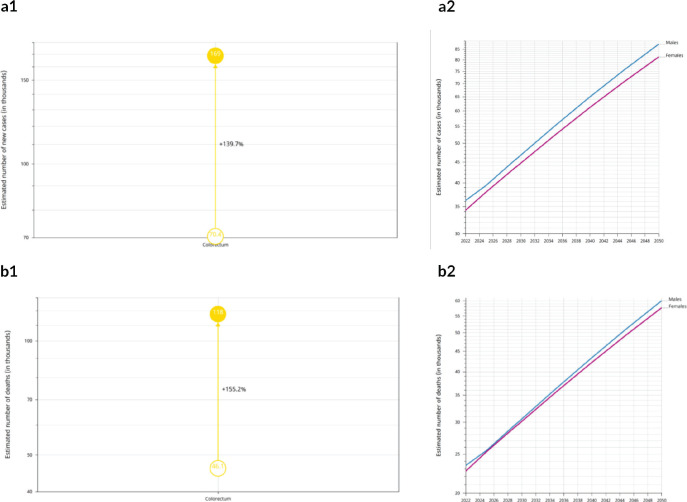
A1 & A2: Estimated number of new cases from 2022 to 2050, both sexes, age [0–85+] Africa. B1 & B2: Estimated number of deaths from 2022 to 2050, Both sexes, age [0–85+] Africa.

In 2022, CRC accounted for 9.6% of all cancers globally and 5.9% of cancers on the African continent Figure 4. At national levels, it ranked in the top four of the most commonly diagnosed cancers in more than half (56%) of African countries. By regional distribution, the highest incidences and mortalities were recorded in northern Africa, Eastern African and some parts of South Africa, while West and Central Africa had the least incidences and mortalities Figure 1.

### Predicted incidence and mortality of CRC in Africa by 2050

**Figure 5.**
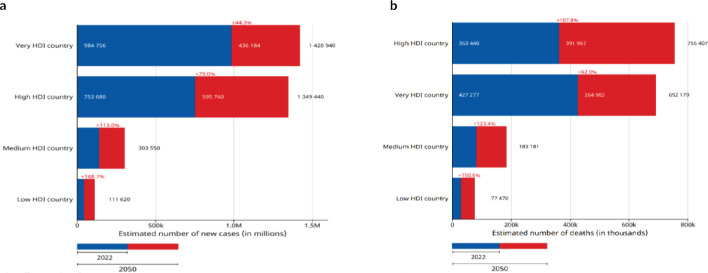
(a): Estimated number of new cases by HDI from 2022 to 2050, both sexes, age (0–85+), colorectum. (b): Estimated number of deaths by HDI from 2022 to 2050, both sexes, Age (0–85+), colorectum.

CRC is predicted to increase from 70,428 in 2022 to 168,700 in 2050 if the current incidence rate is maintained. Similarly, by the current mortality rate, an estimated 117,600 people are expected to die from CRC in 2050 up from 46,061 in 2022 Table 3. These represent percentage increases of 139.7% for incidence and 155.2% for mortality, respectively, Figure 5. By HDI group, the highest absolute increase in new cases and deaths is predicted to occur in high HDI countries with 30.69% more cases (436,184 additional cases) and 51.9% more deaths by 2050. However, the highest relative increases in incidences and mortalities are predicted to occur in low HDI countries, (148.7% and 150.6%, respectively) reflecting the rapidly changing demographics and lifestyles in these counties.

## Discussions

In this study, we assessed the burden and mortality rates of CRC across the African continent as of 2022 and used the incidence rate to predict CRC burden and mortality by 2050. Our findings revealed that the incidence of CRC had doubled (8.2 versus 4.04 per 100,000 persons) compared to a decade ago [15]. We observed a heterogeneous increase in CRC cases where North and East Africa had more than half of the continent’s CRC cases and mortalities. This trend could be attributed to the rapid increase in all the known risk factors for CRC across Africa, i.e., obesity [16], smoking [17], physical inactivity and sedentary lifestyle [18] and high consumption of salt [19]. Also, increased standards of living, especially in transition countries in North Africa have led to more elderly people, sedentary lifestyles and increased adoption of Western diets that are mostly junk foods, all of which lead to increased cases of CRC. It is also true that as diagnosis facilities become widespread on the continent and more countries adopt routine use of cancer registries, more CRC cases will be diagnosed and recorded [20].

A decade later, countries in North Africa and the Indian Ocean islands of Mauritius and Reunion still dominate the top ten countries with the highest burden of CRC in Africa, with ASR for incidence ranging from 11.9% in Tunisia to 17.1% in Algeria. Predictions for 2050 dictate that this trend will remain unless these countries make drastic changes in the modifiable risk factors, diagnosis and treatment of the disease. By demographics, most of the countries in the top ten have majority Arab populations. While it is likely that ethnicity could play a role, there is no genetic or lifestyle evidence to show that being Arab predisposes one to CRC [21, 22]. Analysis of countries in the top and bottom ten reveals a peculiar trend; those in the top ten except Somalia, largely have a better standard of living than their counterparts in the bottom ten. This could be directly associated with the increased prevalence of CRC modifiable risk factors such as sedentary lifestyle, obesity and consumption of junk foods which are all associated with urbanization and increased living standards [23]. Similarly, better healthcare systems, widespread cancer diagnostic facilities and proper record keeping are more prevalent in countries with higher standards of living [24], and so they are more likely to diagnose and report more cases of CRC. This trend should be a warning to the relatively less urbanised countries in the bottom ten, that as they urbanise, health authorities should take precautions to control the lifestyle and dietary risks of CRC associated with urban lifestyle.

Stratification by gender showed a clear predilection for males compared to females. This is consistent with other continents including North America [25], Asia [26] and Europe [27]. This could be attributed to men not generally taken better care of themselves than women. For instance, excess belly fat which is a common occurrence among men is a known risk factor in developing colon cancer as it can surround vital intestinal organs [28]. Future prevention measures should thus take this disparity into consideration and tailor gender-responsive measures. In terms of HDI, current estimates and predictions for 2050 indicate that CRC will be a bigger burden for high HDI countries. However, analysis by percentage/relative increase reveals higher future incidence and mortality rates for low HDI countries. As of 2022, 51% (27) of African countries were categorised as low HDI countries [29]. Therefore, these countries will need to take a critical look at these trends and institute policy adjustments that mitigate these threats. Easily implementable strategies such as enhanced public awareness campaigns and lifestyle education have had significant impacts on cancer prevention in the past [30]. These should be encouraged as governments plan for other cost-intensive interventions.

By 2050, the incidence rate of CRC will more than double across Africa from 70,428 to 174,396 cases. The continent will also experience the highest relative increase in the incidence (147.6%) and mortality (153.8%) of CRC compared to all other continents. These predictions are based on the reports that Africa will be the fastest growing continent in the world in the coming decades [31], with an exponential boom in population, decreased mortality from communicable illness, better diagnostic facilities and increased prevalence of all CRC associated risks that are linked to urbanization. Available evidence suggests that CRC incidence and mortality can be reduced through prevention of the modifiable lifestyle-related risk factors [32, 33], and evidence-based routine screening of at-risk populations. The estimated efficacy of CRC screening ranges from 2.6% (when single screening using fecal occult blood test is done) to over 50% (when colonoscopy is done every 10 years, or a combination of annual fecal occult blood test and sigmoidoscopy every 5 years) [34].

While suggestions based on mathematical modelling have been made for regular colonoscopy to commence at 50 years of age [35], there is a steady rise in the incidence of CRC among young adults across Africa [36, 37]. Wide-scale population-based screening may not be plausible in the face of the high burden of communicable diseases, low human resource capacity, lack of colonoscopy facilities and relatively low burden of CRC compared to other diseases [38]. Therefore, further studies will be needed to guide more accurate recommendations for routine CRC screening on the continent. Countries will also have to make deliberate policy adjustments that help tackle CRC in the presence of a high burden of communicable diseases and other non-communicable illnesses that are all competing for the limited resources available [39]. Strategies such as integrating CRC prevention and treatment into national health policies and cancer control plans to ensure a coordinated and effective response, increased resource allocation to cancer care and prevention programs, training more oncologists, strengthening diagnostic facilities and supporting research on the specific genetic, environmental and lifestyle factors contributing to the rise in CRC in Africa among others, will ensure that CRC is properly tackled.

## Limitations

In this study, while we assumed that national rates of CRC as estimated in 2022 will not change between 2022 and 2050, there is good evidence that incidence rates will change as HDI changes due to regular screening in High HDI countries. Therefore, cancer predictions for future years should be interpreted with due caution. Secondly, although GLOBOCAN datasets provide comprehensive and up-to-date evidence of the global, regional and national burden of the different types of cancers that greatly facilitate planning, policy formulation and research in Africa, the unavailability and poor quality of some of the data sources on the African continent remains a huge challenge. As a result, countries without data may have their national incidence and mortality rates estimated by modelling, using data derived from cancer registries in neighbouring countries. Finally, due to the continuous improvement in the quality and availability of data sources, changes in methodology are sometimes necessary. Consequently, the estimates may not be comparable over time. Thus, it is important to exercise considerable caution when interpreting the current estimates in comparison to those published in previous versions of GLOBOCAN.

## Conclusion

In summary, our findings revealed that both the incidence and mortality of CRC are on the increase in Africa, and will be more than doubled by 2050 if concrete actions are not taken. Modifiable CRC risk factors such as alcohol consumption, smoking, poor nutrition, sedentary behaviour and metabolic syndrome are all on the rise as Africa rapidly urbanises. Therefore, there is a need to put emphasis on primary prevention of modifiable risk factors, and evidence-based early diagnosis and treatment of CRC. Finally, member states need to deliberately improve cancer registries across the continent to capture quality data that offers more accurate predictions for planning, policy formulation and research purposes. Only through a multi-thronged approach can the CRC burden be addressed on the continent.

## Conflicts of interest

The authors declare that they have no competing interests.

## Funding

No external funding was received.

## Ethics approval and consent to participate

NA.

## Author contributions

Study concept and design: MLILV. Analysis and interpretation of data: MLILV. Drafting the manuscript: MLILV. Critical revision of the manuscript for important intellectual content: LL and QZ.

## Figures and Tables

**Table 1.** Estimated number of CRC cases and deaths, and cumulative risks of developing CRC and mortality before 75 years of age across Africa.

	Population		Incidence				Mortality			
	Total (‘000)	Percentage of Africa total (%)	Number of new cases	Percentage of Africa total (%)	Cum. Risk 0–74 (%)	M:F	Number of deaths	Percentage of Africa total (%)	Cum. Risk 0–74 (%)	M:F
Eastern Africa	445,406	33.2	19,370	27.5	0.89	0.94	13 801	30.0	0.63	0.92
Middle Africa	179,595	13.4	5,397	7.7	0.60	1.11	3 840	8.3	0.45	1.10
Northern Africa	246,233	18.4	23,557	33.4	1.20	1.04	13 085	28.4	0.58	1.04
Southern Africa	67,504	5.0	7,707	11.0	1.50	1.04	4 830	10.5	0.82	1.08
Western Africa	401,861	30.0	14,397	20.4	0.75	1.25	10 531	22.8	0.56	1.24
Total numbers	1,340,599	100	70,428	100			46, 087	100		

**Table 2.** Estimated ASRs of CRC by continents.

Continents	ASR incidence (per 100,000)	Cum risk (74 years)	ASR mortality (per 100,000)	Cum risk (74 years)
Asia	15.6	1.8	7.1	0.73
Europe	30.5	3.6	12.1	1.30
North America	27.2	3.1	8.2	0.83
Latin America & the Caribbean	16.9	1.9	8.2	0.88
Africa	8.2	0.94	5.6	0.59
Oceania	31.1	3.4	9.2	0.85

**Table 3.** Estimated number of new CRC cases in 2022 and 2050 predictions.

Cancer site	Total cases in 2022	Predicted cases in 2050	Total deaths in 2022	Predicted deaths in 2050
Colon	36,427	87,200	24,815	63,100
Rectum	27,645	65,800	16,991	43,500
Anus	6,356	15,700	4,281	11,000
Total	70,428	168,700	46,087	117,600
